# An fMRI Study of Neuronal Activation in Schizophrenia Patients with and without Previous Cannabis Use

**DOI:** 10.3389/fpsyt.2012.00094

**Published:** 2012-10-30

**Authors:** Else-Marie Løberg, Merethe Nygård, Jan Øystein Berle, Erik Johnsen, Rune A. Kroken, Hugo A. Jørgensen, Kenneth Hugdahl

**Affiliations:** ^1^Department of Biological and Medical Psychology, University of BergenBergen, Norway; ^2^Division of Psychiatry, Haukeland University HospitalBergen, Norway; ^3^Department of Clinical Medicine, University of BergenBergen, Norway; ^4^Department of Radiology, Haukeland University HospitalBergen, Norway

**Keywords:** schizophrenia, fMRI, cannabis, dichotic listening, default mode network, effort mode network, brain activation, cognitive control

## Abstract

Previous studies have mostly shown positive effects of cannabis use on cognition in patients with schizophrenia, which could reflect lower neurocognitive vulnerability. There are however no studies comparing whether such cognitive differences have neuronal correlates. Thus, the aim of the present study was to compare whether patients with previous cannabis use differ in brain activation from patients who has never used cannabis. The patients groups were compared on the ability to up-regulate an effort mode network during a cognitive task and down-regulate activation in the same network during a task-absent condition. Task-present and task-absent brain activation was measured by functional magnetic resonance neuroimaging (fMRI). Twenty-six patients with a DSM-IV and ICD-10 diagnosis of schizophrenia were grouped into a previous cannabis user group and a no-cannabis group. An auditory dichotic listening task with instructions of attention focus on either the right or left ear stimulus was used to tap verbal processing, attention, and cognitive control, calculated as an aggregate score. When comparing the two groups, there were remaining activations in the task-present condition for the cannabis group, not seen in the no-cannabis group, while there was remaining activation in the task-absent condition for the no-cannabis group, not seen in the cannabis group. Thus, the patients with previous cannabis use showed increased activation in an effort mode network and decreased activation in the default mode network as compared to the no-cannabis group. It is concluded that the present study show some differences in brain activation to a cognitively challenging task between previous cannabis and no-cannabis schizophrenia patients.

## Introduction

It has been shown that cannabis use is more widespread in patients with schizophrenia than in the healthy population (Regier et al., 1990; Arseneault et al., 2004; Barnes et al., 2006). Possibly, cannabis is a risk factor for schizophrenia (Andreasson et al., 1987; Zammit et al., 2002; Arseneault et al., 2004; Moore et al., 2007), mediated by the effect of the main psychoactive ingredient, Delta9-tetrahydrocannabinol (THC) on the endogenous cannabinoid and dopamine systems (D’Souza et al., 2005; Solowij and Michie, 2007; Bossong and Niesink, 2010). Paradoxically, most neurocognitive studies on schizophrenia have shown cannabis use to be a marker of superior performance on neuropsychological tests. A systematic literature review revealed better cognitive functioning in cannabis-using compared to non-cannabis-using patients in a majority of the reviewed 23 studies (Løberg and Hugdahl, 2009). This pattern has been replicated by later studies (DeRosse et al., 2010; Rodriguez-Sanchez et al., 2010), also including two meta-analyses (Rabin et al., 2011; Yucel et al., 2012). It is of importance, however, to differentiate between the consequences of ongoing cannabis use and short-term intoxication effects versus the effects of previous cannabis use as pathway to psychosis (Løberg and Hugdahl, 2009). Recently, a large cross-sectional study compared the short-term effect of cannabis and the effect of life-time cannabis use in 956 patients, and concluded that there was a short- time negative effect on cognition and in contrast a positive long-term effect of life-time use (Meijer et al., 2012). The authors suggested that the life-time cannabis-using group formed a subgroup with a different cognitive profile.

It has been proposed that better cognition reflects lower cognitive vulnerability in schizophrenia patients with a history of cannabis use (Løberg and Hugdahl, 2009). In such a model, deficient cognition is a vulnerability marker, indicating neurodevelopmentally based brain dysfunctions. In the typical patient with no drug use, such brain dysfunctions render individuals susceptible to psychosis. Cannabis use characterizes a subgroup of schizophrenia patients with less cognitive deficits. For these patients, cannabis use disturbs the functional integrity of the brain creating a psychotic breakdown (Løberg and Hugdahl, 2009). Within a stress-vulnerability framework (Zubin and Spring, 1977; Insel, 2010), the tendency to develop schizophrenia is a function of vulnerability × stress. When cannabis is entered into the equation as a stress factor, the importance of a high vulnerability load is decreased. More cannabis/stress × less vulnerability/neurocognitive deficits generates a tendency to develop schizophrenia that is similar to less cannabis/stress × high vulnerability/neurocognitive deficits. Cognition is not an optimal vulnerability marker, however, and neuropsychological test performance is also confounded by motivational, and behavioral variables, and is at best an indirect measure of brain functioning that may vary with fluctuating psychosis symptoms and treatment (Johnsen et al., 2011). It would therefore be of interest to investigate if the observed cognitive differences between cannabis and no-cannabis schizophrenia patients also would be revealed in a difference in brain activation between these sub-groups.

We therefore conducted a functional magnetic resonance imaging (fMRI) study on a group of schizophrenia patients with previous use of cannabis compared with a group of patients without such experience. Of the few previous brain-imaging studies conducted on cannabis use and schizophrenia, firm conclusions cannot be drawn, and even evidence of adverse effects of cannabis on the brain in healthy individuals is inconclusive (Block et al., 2000; Jager et al., 2006; DeLisi, 2008; Martin-Santos et al., 2010). Most functional brain-imaging studies on cannabis and schizophrenia have focused on the immediate and acute effects of cannabis, not relevant for the present study. One fMRI study, however, found that socio-emotional processing was less impaired in patients with a dual diagnosis (mainly cannabis users) than schizophrenia alone (Potvin et al., 2007). Studies comparing schizophrenia patients with and without cannabis use by means of structural MRI and diffusion tensor imaging (DTI) have shown more normalized (Dekker et al., 2010), more anomalous (Szeszko et al., 2007; Bangalore et al., 2008; Rais et al., 2008; Ashtari et al., 2011; Ho et al., 2011; James et al., 2011; Solowij et al., 2011), and equivalent (Block et al., 2000; Cahn et al., 2004; Wobrock et al., 2009; Cohen et al., 2012) brain anatomy in the cannabis group, thus making firm conclusions difficult also when it comes to structural imaging.

Possibly, both the severity and recency of cannabis use influence these results, in addition to interactions with genetic (Ho et al., 2011) and environmental risk factors (Habets et al., 2008). The inconsistency of results may also be attributed to different definitions of cannabis use (current, life-time, or previous use, or a cannabis use disorder). To examine the effects of cannabis on brain functioning within a vulnerability framework, the effects of cannabis use before the development of psychosis is important (Løberg and Hugdahl, 2009). For this reason, only patients with *previous* cannabis use were included in the present study. In addition, this would rule out potential confounding effects of cannabis intoxication or recent cannabis use on brain activation, which have been shown in some studies (Bhattacharyya et al., 2012; Bossong et al., 2012; Skosnik et al., 2012).

Thus, we used an fMRI paradigm comparing activation during alternating cognitive task-present and task-absent conditions. We compared activation in two large-scale cortical networks during these conditions, defined as an effort mode and a default mode (cf. Raichle et al., 2001) network. For the task-present condition a dichotic auditory perception task with attention instructions was chosen (Hugdahl and Andersson, 1986; Løberg et al., 1999). This task has the advantage of simultaneously tapping several cognitive functions that are central to neurocognitive vulnerability in schizophrenia; verbal processing, attention, and cognitive control (Filbey et al., 2008; Wobrock et al., 2009). This also allows for the analysis of aggregate cognition and the interaction between large-scale cortical networks. Thus, the effort mode network was defined as activation in the presence of the task, while the default mode network was defined as activation in the absence of the task. The relationship or anti-correlation between these networks may be of particular interest in clinical groups such as schizophrenia (Broyd et al., 2009; Mannell et al., 2010; Nygård et al., 2012).

The default mode network includes areas in the medial prefrontal and temporal lobes, including orbitofrontal cortex, the posterior cingulate cortex, precuneus (PC), fusiform/lingual gyri, and inferior parietal lobule (Fox and Raichle, 2007; He et al., 2012). It has been suggested that the default mode network reflect endogenous generated thought, e.g., inner speech and self-referential thought (He et al., 2012). The activation of these networks increases during rest and non-task phases, and decreases during periods of goal-directed tasks (Schneider et al., 2011). Several studies have shown that patients with schizophrenia fail to show this brain activation pattern, especially the task-induced de-activation, as compared with healthy controls (Pomarol-Clotet et al., 2008; Whitfield-Gabrieli et al., 2009; Mannell et al., 2010; Schneider et al., 2011; He et al., 2012; Nygård et al., 2012). The effort mode network, on the other hand, is assumed to involve areas in the inferior and middle frontal gyrus, supplementary motor area (SMA), anterior cingulate, posterior temporal cortex, and parietal cortex (cf. Fox and Raichle, 2007; Hugdahl et al., 2009; Nygård et al., 2012). Decreased brain activation to effort demanding tasks have been shown repeatedly in schizophrenia (Hugdahl et al., 2004, 2009; Karlsgodt et al., 2007; Koch et al., 2008; Nygård et al., 2012). Following the paradigms used in the original resting state, default mode, studies (e.g., Buckner et al., 1996; Shulman et al., 1997; see also Binder, 2012 for reviews of the early history of resting state studies), where a “rest” or “passive” task was used to contrast active task processing, we analyzed the OFF-block against ON-blocks to contrast a rest, or passive, task (OFF-blocks) with an active task (ON-blocks). In this way we could study the ongoing interaction between task-absent and task-present state processing activity. This was achieved simply by comparing activation for the ON–OFF-block contrast with activation for the OFF–ON block contrast. This is not the “traditional” way of studying the default mode network activity. However, there are several arguments for the present procedure. In this way it is also possible to study the switching between activation states during task-present and task-absent conditions, and whether one group is impaired compared to another group. The use of a prolonged resting period with no-task activation (as in a typical resting state paradigm) has the disadvantage that two states are not studied simultaneously.

The aim of the present study was therefore to examine brain activation in patients with schizophrenia with and without a history of previous cannabis use, and to explore if previous neurocognitive differences between these groups have neuronal substrates that could be detectable in an fMRI study. We were particularly interested in differences in the ability to up-regulate the effort mode network during the task-present condition.

## Materials and Methods

### Subjects and clinical assessments

Thirty-one patients with a DSM-IV (American Psychiatric Association, 1994) and ICD-10 (WHO, 1992) diagnosis of schizophrenia were included in the study, diagnosed by means of Structural Clinical Interview for DSM-IV (First et al., 1995). Five of these patients were subsequently excluded due to sporadic cannabis use that was difficult to categorize. The remaining 26 patients were divided into two sub-groups; a Can− group (*n* = 13) with no history of cannabis use and a Can+ group (*n* = 13) with a history of cannabis use. There were 8/5 and 11/2 men/women in the two groups, respectively. Clinical, cognitive, and demographic data, as well as data on social and general functioning (Birchwood et al., 1990), is presented in Table 1.

**Table 1:** **Demographic, clinical, and cognitive data by group**.

	No-cannabis group (Can−)		Cannabis group (Can+)		*P*
	Mean (SD)	Min−Max	Mean (SD)	Min–Max	
Age (years)	36.23 (11.00)	18–57	33.38 (10.36)	19–55	NS
Education (years)	14.04 (3.14)	10–19	11.46 (1.90)	9–15	0.02
Age of onset (years)	25.68 (6.86)	17–39	19.46 (7.00)	7–29	0.04
Duration of illness (years)	11.68 (10.95)	2–33	13.38 (10.68)	4–34	NS
Medication (DDD)	1.42 (0.84)	1–3	1.26 (0.95)	0–3	NS
**PANSS**					
Total scores	52.83 (15.67)	34–81	49.15 (12.19)	31–75	NS
Positive subscale	11.83 (5.89)	7–22	12.54 (5.83)	7–23	NS
Negative subscale	17.25 (6.12)	10–31	13.23 (5.40)	7–26	NS
Gen. psychopath. subscale	23.75 (7.10)	16–37	23.38 (6.61)	16–40	NS
GAF function scores	44.73 (19.51)	20–80	44.00 (16.65)	28–81	NS
**SOCIAL FUNCTIONING SCALE**					
Withdrawal	101.94 (6.70)	90.50–110.00	107.00 (8.35)	93. 50–116.50	NS
Interpersonal behavior	117.67 (17.11)	96.00–145.00	121.56 (19.89)	100.00–145.00	NS
Prosocial activities	112.39 (12.17)	92.00–124.00	102.56 (14.14)	73.50–118.50	NS
Recreation	110.33 (17.04)	80.00–133.00	112.31 (13.63)	96.00–135.00	NS
Independence-Competence	106.83 (14.49)	79.00–127.00	109.00 (10.82)	93.50–128.00	NS
Independence-Performance	113.89 (10.61)	97.50–123.00	108.13 (13.15)	95.50–123.00	NS
Employment/Occupation	106.06 (14.79)	81.50–122.50	99.44 (14.59)	81.50–122.50	NS
**COGNITIVE FUNCTIONING**					
Verbal abilities	48.92 (5.25)	39.68–58.52	51.57 (4.87)	43.81–59.08	NS
Visuospatial abilities	41.24 (8.67)	26.64–50.82	47.25 (11.07)	22.94–58.86	NS
Learning	40.18 (8.18)	30.75–56.58	46.44 (11.81)	33.88–64.58	NS
Memory	43.41 (6.67)	35.71–54.18	48.92 (7.14)	38.86–61.66	NS
Attention/working memory	42.12 (8.34)	31.52–55.29	44.68 (6.75)	33.77–51.34	NS
Executive functioning	37.48 (15.89)	18.35–64.22	40.48 (17.51)	2.51–56.87	NS
Visuomotor speed	37.79 (5.80)	31.66–47.15	42.27 (6.40)	33.40–52.32	NS

*SD, standard deviation; Age of onset, age at first psychotic experience; Duration of illness, age at testing minus age of onset; DDD, defined daily dose; PANSS, The Positive and Negative Syndrome Scale for Schizophrenia; GAF, Global Assessment of Functioning Scale. The seven cognitive domains signify mean *t*-scores. Verbal abilities = WAIS-(Wechsler Adult Intelligence Scale) III Similarities, WAIS-III Vocabulary, Controlled oral word association test Letters/animals. Visuospatial abilities = WAIS-III Digit Symbol-coding, WAIS-III Block design, Rey–Osterrieth Complex Figure test Copy. Learning = The California Verbal Learning Test-II (Immediate Recall), WAIS-Digit Span. Memory = CVLT-II (Delayed Recall), ROCF Delayed recall. Attention/working memory = WAIS-III Digit Span, Digit Vigilance test, CalCAP Continuous Performance test (Choice Reaction Time/Sequential Reaction Time), Trail Making test B. Executive functioning = Wisconsin Card Sorting test, Stroop color/word conflict. Visuomotor speed = Trail Making test A, WAIS-III Digit Symbol-coding, Grooved pegboard test, CalCAP Reaction time test. There were missing data for the following variables: Education (2), Medication (7), Duration of illness (3), Age of onset (3), PANSS (1), Cognition (7), Social functioning (8), and GAF (4)*.

History of cannabis use was based on clinical records and SCID-interviews, and was further validated through a questionnaire specifically designed for this study. The questionnaire was filled out twice; by the responsible clinician (psychiatrist/psychologist) and a research psychologist. Lifetime and current illegal drug use was recorded, including types of drugs, how often and over how long period they had been used. The patients that reported regular cannabis use over at least 2 years or frequent (at least weekly) cannabis use over 6 months were included in the Can+ group. Patients with no history of cannabis use were included in the Can− group. Sporadic use of amphetamine, LSD, and ecstasy were reported for 5, 3, and 1 of the Can+ patients, respectively. For all Can+ patients, except one, cannabis use started before their psychosis debut, in the age range of 11–25 years. Exclusion criteria were clinically significant neurological disease, history of head injury, substance abuse within the past 6 month (except two patients who had used cannabis twice the last 6 months, but not the last 2 months), and hearing impairment, defined as failing to correctly perceive tones at 20 dB at 500, 1000, 2000, and 4000 Hz or having an inter-aural difference larger than 15 dB. The Positive and Negative Syndrome Scale (SCI-PANSS; Kay et al., 1989) was used for symptom ratings, performed about half an hour before the MR-scanning. All subjects were right-handed as determined by a self-report questionnaire (Raczkowski et al., 1974). The project was approved by the Regional Committee for Medical Research Ethics at Western Norway Health Authority (REK-Vest).

### Task

The patients were scanned while listening through headphones to dichotic presentations of series of consonant-vowel (CV) syllables. The syllables consisted of the six stop-consonants paired with the vowel/a/to form six CV-syllables/ba/, /da/, /ga/, /ka/, /pa/, /ta/. The CV-syllables were presented through MR compatible headphones with insulating materials that also compensated for the ambient scanner noise. Instructions were given verbally before the experiment and in written form via LCD goggles (NordicNeuroLab)^1^ during the MR-scanning. The patients were instructed to report the syllable they heard best on each trial, and for 2/3 of the trials (pseudo-randomized) they were told to focus on and report only from the right or left ear to increase the attention and executive load of the task (Hugdahl and Andersson, 1986). Which ear to focus attention on and report from was indicated by an arrow in the LCD goggles in addition to the written instructions. Further details about the paradigm are described elsewhere (van den Noort et al., 2008). The verbal response after each syllable pair presentation was recorded with an in-house built air-conducting microphone that was placed on the head-coil and attached to a digital recorder (M-audio Microtracker 24/96^2^, or on a DAT recorder) outside the MR chamber, and the responses were later scored. In order to focus on the cognitive aspects of the dichotic listening (DL) task, we calculated an aggregate score based on the two conditions when subjects are explicitly instructed to focus attention on either the right or left ear stimulus.

### MR-scanning

MR imaging was performed with a 3.0T GE Signa HDx scanner. Head movements were restrained by additional padding inside the head-coil. For positioning the slices for functional imaging parallel to the AC-PC line, a high-resolution T1-weighted 3D volume image was acquired prior to the EPI image acquisitions using a FSPGR pulse sequence with 122 sagittal slices (64 × 64 matrix size, 1.0 mm slice thickness, TE = 30 ms, TR = 1500 ms, FA = 90). The fMRI part involved a sparse-sampling EPI sequence protocol (van den Noort et al., 2008) where the EPI volumes were acquired with repetition time TR = 5.5 s, and acquisition time TA = 1.5 s, with a silent gap of 4 s, during which the CV-syllable stimuli were presented and the verbal responses were recorded. The stimulus (CV-syllables) presentation started 0.6 s after the TA, leaving approximately 2.9 s for verbal responses after each stimulus presentation. A block design with nine ON–OFF-block combinations was used. In total, 184 BOLD sensitive EPI volumes were acquired with 3.44 mm × 3.44 mm × 5.5 mm voxel size and 25 axial slices covering most of the cerebrum. The first four EPI volumes were discarded prior to the processing of the data. There was a MR scanner upgrade about half-way into the project that was outside of our control. This affected the sensitivity in the images. However, there were about equally as many patients before and after the upgrade and a Chi-square test showed that the difference in number of patients in the Can− (before upgrade 7/after upgrade 6) and Can+ (before upgrade 8/after upgrade 5) groups before and after the upgrade was not significant, χ^2^(1) = 0.161, *p* = 0.691, n.s.

### fMRI analysis

The MR DICOM images were converted to the ANALYZE file format using the nICE software version 2.3.6^3^. The converted images were pre-processed using the Statistical Parametric Mapping (SPM8) software package (Wellcome Trust Centre for Neuroimaging)^4^ implemented in Matlab R2009b (Mathworks Sherborn, MA, USA)^5^ and then realigned and corrected for possible movement distortions (unwarp) and normalized into the Montreal Neurologic Institute (MNI) reference brain space (Ashburner and Friston, 1999). The EPI template that is included in the SPM8 software was used for the normalization. The normalized images were re-sampled with an isotropic voxel size of 3 mm × 3 mm × 3 mm and smoothed with an 8 mm full-width at half maximum Gaussian kernel in the *x*, *y*, and *z* directions. The predictors were convoluted with the hemodynamic response function (hrf) and a temporal high pass filter (cut-off: 128 s) was applied, and with a significance threshold of *p* = 0.001.

For the group analyses, the individual contrast-files were subjected to *t*-tests. At the second-level, group analyses, the individual contrast images for the three attention instruction conditions were merged into an aggregated contrast image, subjected to one-sample *t*-tests for main-effects for the groups together and for the respective group separately, and to two-sample *t*-tests for comparisons between the groups. The results were explored at an FWE corrected statistical threshold of *p* < 0.05 for an omnibus analysis involving both groups together, and by an uncorrected threshold of *p* < 0.001 when analyzing the groups separately and compared with each other, due to loss of statistical power. Only clusters with at least 10 (FEW corrected analysis) or 20 (uncorrected analyses) voxels were considered. Coordinates in Montreal Neurological Institute (MNI) space were validated for anatomical localization using the Automated Anatomical Labeling (AAL; Tzourio-Mazoyer et al., 2002) template in the MRIcron software^6^.

The task condition, when the syllables were presented, corresponded to the ON-blocks condition. The no-task condition, when no stimuli were presented, corresponded to the OFF-blocks condition. There were three different instructions (conditions) of how to focus attention, each with a set of 30 syllables, yielding 90 syllable pair presentations. Each ON-block consisted of 10 syllable pair presentations with a unique instruction for each ON-block, with three repetitions of each instruction condition. Thus, there were a total of nine ON-blocks that were alternated with nine OFF-blocks with no stimuli presentations. Within each ON-block, the inter-stimulus interval was 5.5 s, and the length of an ON-block and corresponding OFF-block was 65 s. To test the ability to up-regulate and down-regulate activation as hypothesized the change in activation between the task-present and task-absent conditions were examined by using contrasts comparing activation from the ON and OFF-blocks.

In order to separate significant clusters being activated when stimuli were presented (task condition), from clusters being activated in the absence of stimuli (no-task condition), respectively, two different approaches to the data were used. The first approach would correspond to setting up contrasts with images acquired during ON-blocks minus images acquired during OFF-blocks. The second approach would correspond to setting up contrasts with images acquired during OFF-blocks minus images acquired during ON-blocks. Thus, voxels activated during ON–OFF-blocks, and OFF–ON-blocks, respectively would by definition be separate activations, not occurring at the same time, and following different time-courses dependent on whether a stimulus was presented or not. Thus, the ON–OFF contrast = the average of activations from images acquired during the ON-blocks. The OFF–ON contrast = the average of activations from images acquired during the OFF-blocks.

**Figure 1:**
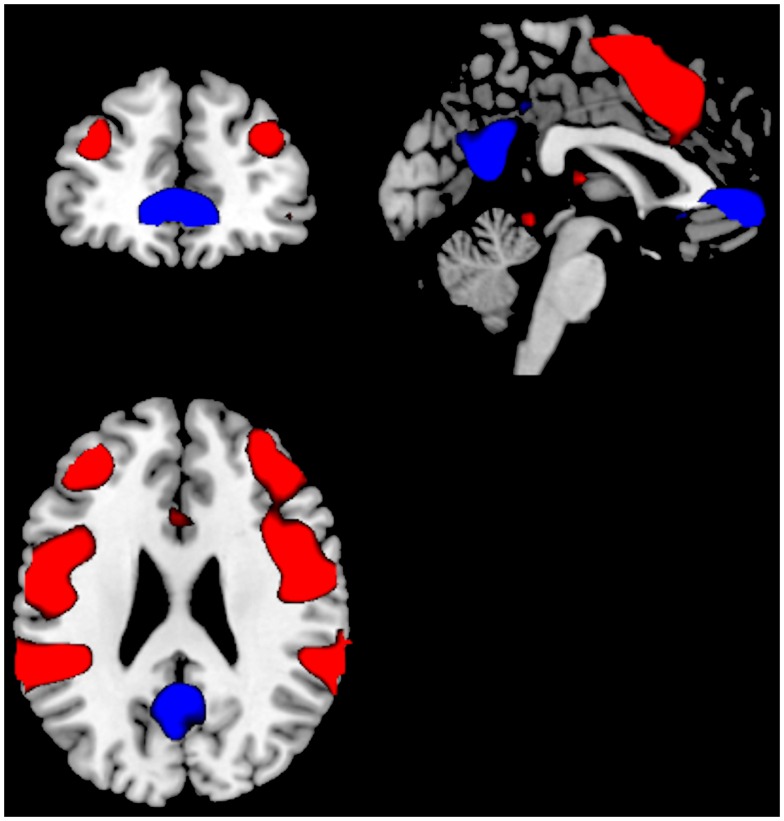
**Significant activations for the main-effect of ON–OFF during task-presence (red color), and OFF–ON during task-absence (blue color), for both groups together**. Activations were thresholded at an FEW correction of *p* < 0.05 and with a minimum of 10 voxels to define a cluster.

**Figure 2:**
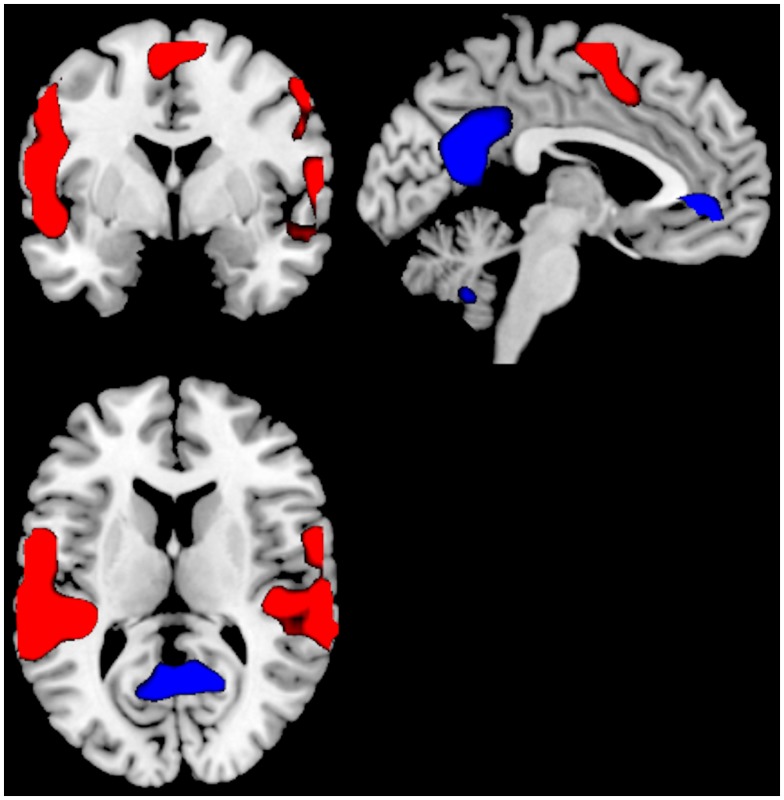
**Significant activations for the main-effect of ON–OFF during task-presence (red color), and OFF–ON during task-absence (blue color), for the Can− group**. Activations were thresholded at uncorrected *p* < 0.001 and with a minimum of 20 voxels to define a cluster.

**Figure 3:**
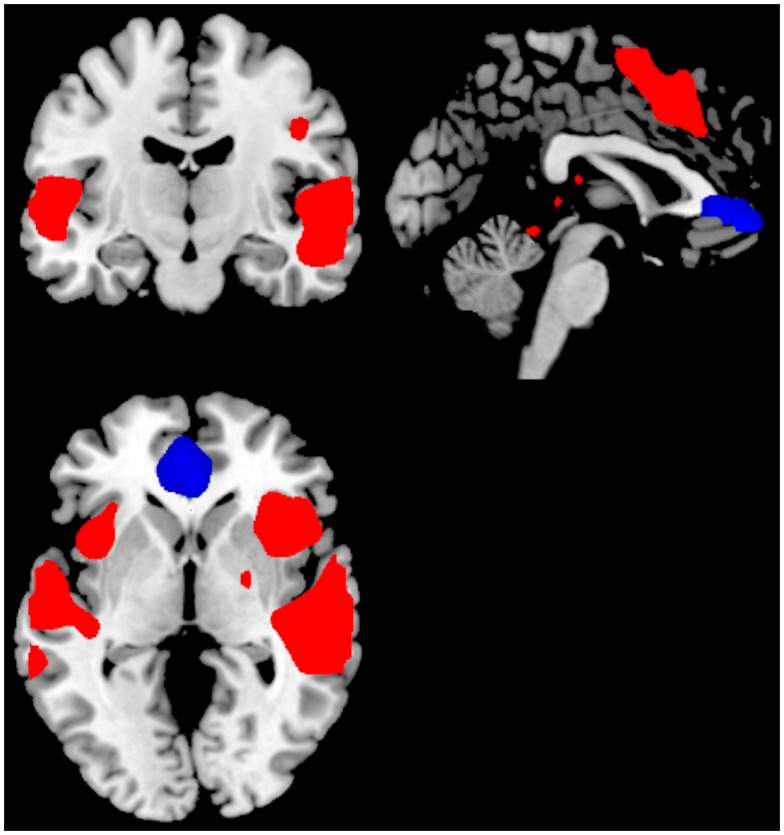
**Significant activations for the main-effect of ON–OFF during task-presence (red color), and OFF–ON during task-absence (blue color), for the Can+ group**. Activations were thresholded at uncorrected *p* < 0.001 and with a minimum of 20 voxels to define a cluster.

**Figure 4:**
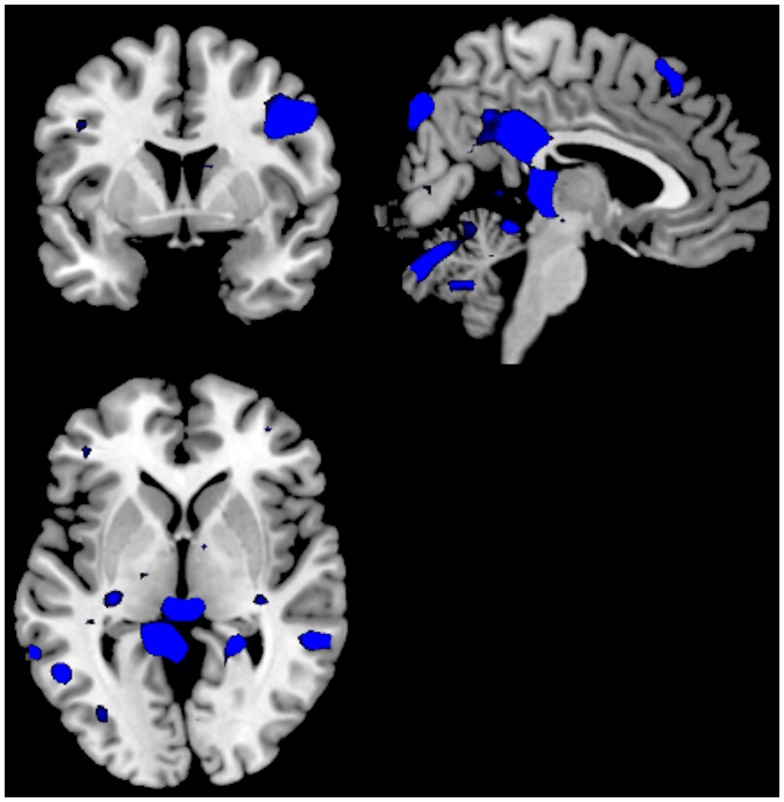
**Significant activations for the ON–OFF (red colors) and OFF–ON (blue colors) contrasts when comparing the groups against each other; Can− minus Can+**. The absence of any red colored activations indicate absence of significant activations for the ON–OFF comparison. Activations were thresholded at uncorrected *p* < 0.001 and with a minimum of 20 voxels to define a cluster.

**Figure 5:**
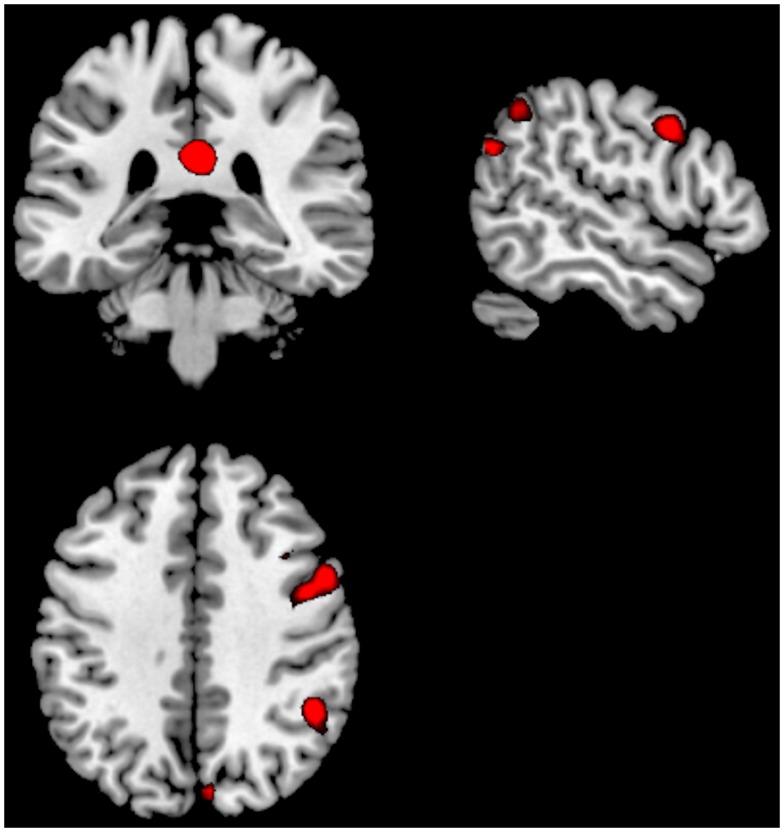
**Significant activations for the ON–OFF (red colors) and OFF–ON (blue colors) contrasts when comparing the groups against each other; Can+ minus Can−**. The absence of any blue colored activations indicate absence of significant activations for the OFF–ON comparison. Activations were thresholded at uncorrected *p* < 0.001 and with a minimum of 20 voxels to define a cluster.

Significant voxels being activated during the ON time-course would broadly speaking belong to task-present, or effort mode network (van Wageningen et al., 2009), while significant voxels being activated during the OFF time-course would broadly speaking belong to task-absent, or default mode network (Raichle et al., 2001; Fox and Raichle, 2007). Significant clusters have been plotted as coronal, sagittal, and axial slices in Figures 1–5. This means that some clusters may not be seen in all slices. All significant clusters are however specified in Table 2 as MNI coordinates for the respective activated clusters, and in the text. All figure displays are with neurological display convention, i.e., left is left and right is right.

**Table 2:** **Anatomical localization, MNI *X*, *Y*, *Z* coordinates, *Z*-value, and corresponding figures for the different contrasts tested**.

Comparison/coordinates	*X*	*Y*	*Z*	*Z*-value	Corresponding figure
**GROUPS TOGETHER**					
**ON–OFF**					Figure 1
Right middle temporal gyrus	63	−22	−2	7.42	
Left superior temporal gyrus	−63	−13	4	6.95	
Supplementary motor area/	0	−1	64	6.51	
Anterior cingulate cortex	1	22	29	5.89	
Left inferior parietal lobule	−45	−58	55	5.50	
Right inferior parietal lobule	51	−55	55	5.35	
Left postcentral gyrus	−57	−10	43	7.08	
Left middle frontal gyrus	−36	44	25	4.77	
Right middle frontal gyrus	36	41	28	4.53	
**OFF–ON**					
Right medial orbitofrontal cortex	0	44	−5	5.43	
Left calcarine/precuneus	−3	−58	16	4.50	
Right occipital lobe	6	−55	16	4.50	
Posterior cingulate cortex	0	−52	28	4.47	
**GROUPS SEPARATED**; **Can**−					
**ON–OFF**					Figure 2
Right superior temporal gyrus	60	−19	−5		
Left middle temporal gyrus	−60	−22	−2		
Right supplementary motor area	6	−4	64		
Left inferior parietal lobule	−48	−55	55		
Right inferior parietal lobule	48	−55	52		
**OFF–ON**					
Right precuneus	6	−52	28		
Left anterior cingulate	−6	35	−2		
Medial orbitofrontal cortex	0	44	−5		
**GROUPS SEPARATED; Can+**					
**ON–OFF**					Figure 3
Right superior temporal gyrus	60	−25	1	5.52	
Left middle temporal gyrus	−60	−10	−5	5.48	
Right supplementary motor area	6	2	64	4.99	
Cerebellum	0	−40	−8	4.54	
**OFF–ON**					
Left medial orbitofrontal gyrus	0	44	−8	3.67	
**GROUPS COMPARED; Can**−**minus Can+**					
**ON–OFF**					Figure 4
No sign. activations					
**OFF–ON**					
Right posterior cingulate cortex	3	−37	28	4.23	
Right inferior parietal lobule	48	−49	43	4.17	
Right occipital lobe	6	−82	43	3.75	
Left cerebellum	−33	−79	−35	3.74	
Right precentral gyrus	51	8	37	3.68	
Left superior temporal gyrus	−45	−28	10	3.63	
Left inferior frontal gyrus	−51	32	22	3.49	
Right angular gyrus	54	−64	28	3.82	
Left lingual gyrus	−9	−43	4	3.75	
Right precuneus	6	−82	43	3.75	
**GROUPS COMPARED; Can+ MINUS Can**−					
**ON–OFF**					Figure 5
Right posterior cingulate cortex	3	−34	25	3.99	
Right inferior parietal lobule	48	−49	43	3.59	
Right precentral gyrus	51	8	37	3.46	
**OFF–ON**					
No sign. activations					

### Behavioral, clinical, and demographic data analysis

Group differences for the clinical, cognitive, and demographic data and averaged scores from the DL task that are reported in Table 1 were tested by means of one-way ANOVAs and for gender by means of Chi-square. The DL performance data were scored and summed for right and left correct ear reports and then averaged across the two attention instruction conditions for each subject. Two kinds of aggregate DL scores were calculated. One involved averaging the right ear score when instructed to focus attention to the right side and the left ear score when instructed to pay attention to the left side. The other aggregate was taking both the right and left ear scores together, for both attention instruction conditions. In order to explore whether percentage of correct reports on the cognitive DL task was associated with increased or decreased brain activation in key areas in the default and effort mode networks, we correlated the fMRI BOLD response in the combined area of SMA and anterior cingulate cortex (ACC), and in the Precuneus (PC) area with the aggregated percentage correct reports in the DL task, respectively, and separated for the two groups. These brain regions were defined as regions of interest (ROIs) because they are key regions in the effort mode, and default mode networks, respectively. The ROIs were defined anatomically from the automated anatomical labeling (AAL), Tzourio-Mazoyer et al., 2002) atlas for each subject, to avoid confounding with individual activations, and the averaged BOLD data were then extracted for each subject across the time-series of image acquisitions.

## Results

### Clinical, demographic data

There were no significant differences between the two groups for the clinical, DL, cognitive, and demographic data, the social or general functioning data shown in Table 1, expect for Education [*F*(1, 22) = 5.93 *p* = 0.02] and Age of onset [*F*(1, 22) = 4.62 *p* > 0.04]. The Can+ group had fewer years of education, and an earlier age of illness onset.

### fMRI data

#### Groups together

See Table 2 for MNI coordinates for the respective activated clusters. A first analysis compared the main-effect of activations during task processing (ON-blocks) and during task-absence (OFF-blocks), across the two groups.

The ON–OFF contrast showed significant clusters in the left and right superior and middle temporal gyrus, in the SMA, and extending into the ACC, left and right inferior parietal gyrus, left postcentral gyrus, left and right middle frontal gyrus. Thus, the activations seen for the ON–OFF contrast, across groups were essentially located in the effort mode network and adjacent areas.

The OFF–ON contrast showed significant clusters in the right medial orbitofrontal cortex, the left PC, right PC/calcarine sulcus, posterior cingulate cortex. See Figure 1 and Table 2. Thus, the activations seen for the OFF–ON contrast, across groups were essentially located in the default mode network.

#### Groups separated

A second analysis compared the main-effect of activations during task-presence (ON-blocks) and during task-absence (OFF-blocks), separate for the two groups. For the Can− group, the ON–OFF contrast showed significant clusters in the right superior and left middle temporal gyri, SMA, left and right inferior parietal lobules. The corresponding OFF–ON contrast showed significant clusters in the right PC, left ACC, and extending into medial orbitofrontal cortex. See Figure 2 and Table 2.

For the Can+ group the ON–OFF contrast showed significant clusters in the right superior temporal gyrus, left middle temporal gyrus, right SMA, extending into the ACC, and the cerebellum. The corresponding OFF–ON contrast showed significant clusters in the right medial orbitofrontal cortex. See Figure 3 and Table 2.

#### Groups compared

A third analysis compared the two groups in the same analysis, separately for ON–OFF and OFF–ON contrasts. For the Can− minus Can+ ON–OFF comparison there were no remaining significant clusters. For the OFF–ON comparison there were significant clusters in posterior cingulate cortex, left inferior frontal gyrus, right inferior parietal lobule, right precentral gyrus, right occipital lobe, left superior temporal lobe. See Figure 4 and Table 2.

For the as Can+ minus Can− ON–OFF comparison there were significant clusters in the right posterior cingulate cortex, right inferior parietal lobule, the right precentral gyrus. For the OFF–ON comparison there were no remaining significant clusters. See Figure 5 and Table 2.

#### DL performance data and correlations with BOLD data

The mean correct reports for the DL aggregate score based on the single right and left ear score for the attend-right and attend-left instruction, respectively, were; 34.35% (8.83) and 43.20 (8.93) for the Can− and Can+ groups, respectively. An ANOVA showed the mean difference to be significant, *F*(1, 24) = 6.44, *p* = 0.018. However, taking also the overall percentage correct reports into consideration, i.e., including also the correct reports from the non-attended ear, the significance disappeared, *F*(1, 24) = 2.16, *p* = 0.154, n.s. The means for the overall aggregate scores were; 34.55 (6.79) and 38.39 (6.53) for the Can− and Can+ groups, respectively. There were no significant correlations for the selected BOLD ROIs and DL mean scores, neither for the Can−, nor for the Can+ group, or for the single or overall DL aggregate scores.

## Discussion

Although the Can− and Can+ group showed substantial similarities across activation patterns, there were also group differences in activation and performance, suggesting a difference in neuronal dynamics between the groups. The Can− and the Can+ group showed overlapping activations in the ACC in both conditions, in addition to the expected patterns for the default and effort mode networks. The groups differed, however, in that the intensity and extension of the activations were more pronounced for the task-present condition in the Can+ group, while it was more pronounced for the task-absent condition in the Can− group (see Figures 2 and 3). These overall differences were further substantiated in the direct comparison between the groups. The Can− group showed no remaining activations in the task-present condition above and beyond what was seen in the Can+ group. For the task-absent condition the Can− group showed activation in the posterior cingulate, PC, inferior parietal lobule, middle temporal gyrus, occipital lobe, which are areas located in the default mode network, while the Can+ group did not show any remaining activation in this condition (see Figures 4 and 5). Thus, the Can+ group showed increased activation in the task-present condition and decreased activation in the default mode network in the absence of the task as compared to the Can− group.

These cortical responses were, to some extent corroborated in the behavioral data, when comparing the groups on single DL aggregate score, although this significance disappeared for the overall aggregate score. There were moreover no significant correlations with the BOLD activation in the selected SMA/ACC and PC ROIs. Thus, although there were some indications of a difference in cortical dynamics and cognitive performance between the groups, in favor of the Can+ groups, it is difficult to draw any firm conclusions from this since the correlations with performance on the cognitive task were not significant. Possibly the fMRI paradigm may detect more subtle group differences than the DL test, in particular for small sample sizes. The limited sample size may be too small to detect differences in DL performance. This is consistent with that the cannabis users showed superior performance on all cognitive domains, but it did not reach significance.

The Can+ group up-regulated the effort mode network during the task-present condition and down-regulated the default mode network during the task-absent condition to a larger extent than the Can− group. This is consistent with other studies showing aberrant activation patterns in schizophrenia patient groups in general. In line with this, both decreased brain activation to effort demanding tasks and increased resting state brain activation have been shown in schizophrenia (Hugdahl et al., 2004; Karlsgodt et al., 2007; Broyd et al., 2009; Whitfield-Gabrieli et al., 2009). Moreover, it has been proposed that hyper-activation of the default brain network during task processing may contribute to thought disturbances and auditory hallucinations in schizophrenia (Whitfield-Gabrieli et al., 2009; Northoff and Qin, 2011). Even though there was some overlap between the patient groups, the Can− group did show a pattern closer to the typical schizophrenia findings, probably indicating more impaired brain functioning.

The BOLD group differences could not be explained by differences in clinical variables. The Can+ group had fewer years of education and earlier age of onset, representing a disadvantage for the Can+ group. The fewer years of education is a paradox, however, since it seemingly suggests worse cognitive functioning, and this is not reflected in the behavioral data by the DL test. Alternatively, it can be attributed to the negative effects of drug use on school performance. It can also be argued that results reflect better social and organizational skills necessary to buy cannabis. This would be consistent with the cannabis users being a better functioning subgroup. But, it seems unlikely that this explanation of the findings, since it does not explain cannabis as a risk factor for schizophrenia and the earlier onset age in the cannabis users. Furthermore, some clinical data from the present study, although limited by small sample size, suggests that the groups did not differ in social functioning. The cannabis users showed better performance on all cognitive domains, however, it should be noted that these differences were not significant. The interpretation and generalizability of the present findings is limited by the small sample, and effects of previous use of other illegal drugs cannot be ruled out. The present sample did not include meth-amphetamine, cocaine, or opiate abusers, and for those that did report use of other drugs, cannabis had nevertheless been the main drug of choice.

The present fMRI activation results are consistent with previous neuropsychological findings with regard to cognition (Løberg and Hugdahl, 2009). Since THC causes adverse cognitive short-term effects in vulnerable individuals (D’Souza et al., 2005), cannabis may cause a *transient* cognitive breakdown enabling a psychotic outbreak, but without the typical long-lasting neurocognitive vulnerability. These changes may be more biochemical in nature, influencing the reactivity of the cannabinoid receptor systems via the cannabinoid receptor 1 (CNR1; DeLisi, 2008; Ho et al., 2011). This receptor system is widely expressed in the brain, especially in areas relevant to schizophrenia (Ho et al., 2011). Furthermore, the developing adolescent brain may be particularly sensitive for these effects (Ho et al., 2011). The notion of a transient change of brain functioning is also supported by findings of fewer neurological soft signs in schizophrenia with cannabis use (Bersani et al., 2002; Stirling et al., 2005; Ruiz-Veguilla et al., 2009). Furthermore, a different illness pathway to schizophrenia is supported by the present findings in the sense that most of the patients started using cannabis before their psychotic debut, in addition to the earlier age of onset in the Can+, which also replicate previous studies (Stirling et al., 2005; Barnes et al., 2006; Large et al., 2011). Taken together, although there were similarities in behavioral responses, there were also differences between the groups in the dynamics of cortical responding, in particular up- and down-regulation of task-present and task-absent-related cortical networks. Future studies should aim at unraveling the exact nature of these differences.

## Conflict of Interest Statement

Author Erik Johnsen has received honoraria for lectures given in meetings arranged by Bristol-Myers Squibb, Eli Lilly, and AstraZeneca, has consulted for Eli Lilly, and has been reimbursed by Eli Lilly and Janssen Cilag for attending conferences. Author Hugo A. Jørgensen has consulted for AstraZeneca and Eli Lilly, and has been reimbursed by Eli Lilly for attending a conference. Author Jan Øystein Berle has received honoraria for lectures given in meetings arranged by Bristol-Myers Squibb, Eli Lilly, Novartis, and AstraZeneca, and has consulted for Eli Lilly. Author Kenneth Hugdahl has shares in the NordicNeuro Lab, Ltd., which supplied the headphones, LCD goggles and response grips for the fMRI scanning. All other authors declare that they have no conflicts of interest.

## Footnotes

^1^http://www.nordicneurolab.no

^2^http://www.m-audio.com

^3^http://www.nordicimaginglab.no

^4^http://www.fil.ion.ucl.ac.uk

^5^http://www.mathworks.com

^6^http://www.mccauslandcenter.sc.edu/mricro/mricron/index.html
