# Corrigendum: An fMRI Study of Neuronal Activation in Schizophrenia Patients with and without Previous Cannabis Use

**DOI:** 10.3389/fpsyt.2013.00051

**Published:** 2013-06-07

**Authors:** Else-Marie Løberg, Merethe Nygård, Jan Øystein Berle, Erik Johnsen, Rune A. Kroken, Hugo A. Jørgensen, Kenneth Hugdahl

**Affiliations:** ^1^Department of Biological and Medical Psychology, University of BergenBergen, Norway; ^2^Division of Psychiatry, Haukeland University HospitalBergen, Norway; ^3^Department of Clinical Medicine, University of BergenBergen, Norway; ^4^Department of Radiology, Haukeland University HospitalBergen, Norway

p. 4, “MR-SCANNING” section, line 7 from bottom:

**The following sentence**

“[ … ] a high-resolution T1-weighted 3D volume image was acquired prior to the EPI image acquisitions using a FSPGR pulse sequence with 122 sagittal slices (64 × 64 matrix size, 1.0 mm slice thickness, TE = 30 ms, TR = 1500 ms, FA = 90). [ … ]”

**should be replaced by**

“[ … ] a high-resolution T1-weighted 3D volume image was acquired prior to the EPI image acquisitions using a FSPGR pulse sequence with 180 sagittal slices (256 × 256 matrix size, field of view (FoV) = 256 mm× 256 mm, 1.0 mm slice thickness, TI = 500 ms, FA = 11). [ … ]”

